# Effects of inspiratory muscle training in patients with hypertension: a meta-analysis

**DOI:** 10.3389/fcvm.2023.1113509

**Published:** 2023-05-23

**Authors:** ShuQi Zheng, Qi Zhang, ShuiYan Li, Shilin Li, Qiuru Yao, Xiaoyan Zheng, Gege Li, Yuting Zeng, Ling Chen, Shuping Chen, Longlong He, Jihua Zou, Qing Zeng

**Affiliations:** ^1^ School of Rehabilitation Sciences, Southern Medical University, Guangzhou, China; ^2^ Department of Rehabilitation Medicine, Zhujiang Hospital, Southern Medical University, Guangzhou, China; ^3^ School of Nursing, Southern Medical University, Guangzhou, China; ^4^ Faculty of Health and Social Sciences, The Hong Kong Polytechnic University, Hong Kong, China

**Keywords:** hypertension, blood pressure, inspiratory muscle training, IMT, meta—analysis

## Abstract

**Objective:**

To explore the effects of inspiratory muscle training (IMT) on hypertension and provide guidance for its clinical application as an auxiliary approach.

**Methods:**

Articles published prior to July 2022 were searched in Cochrane Library, Web of Science, PubMed, Embase, CNKI, and Wanfang databases. Included were randomized controlled studies that used IMT to treat individuals with hypertension. The mean difference (MD) was computed using the Revman 5.4 software. In individuals with hypertension, the effects of IMT on systolic blood pressure (SBP), diastolic blood pressure (DBP), heart rate (HR), and pulse pressure (PP) were compared and studied.

**Results:**

There were found to be eight randomized controlled trials totaling 215 patients. According to a meta-analysis, the IMT reduced the SBP (MD: −12.55 mmHg, 95% CI: −15.78, −9.33), DBP (MD: −4.77 mmHg, 95% CI: −6.00, −3.54), HR (MD: −5.92 bpm, 95% CI: −8.72, −3.12), and PP (MD: −8.92 mmHg, 95% CI: −12.08, −5.76) in patients with hypertension. In subgroup analyses, low-intensity IMT showed a better reduction in SBP (MD: −14.47 mmHg, 95% CI: −17.60, −11.34), DBP (MD: −7.70 mmHg, 95% CI: −10.21, −5.18).

**Conclusion:**

IMT may become an auxiliary means to improve the four hemodynamic indexes (SBP, DBP, HR and PP) in patients with hypertension. In subgroup analyses, low-intensity IMT was more effective in regulating blood pressure than medium-high-intensity IMT.

**Systematic Review Registration:**

https://www.crd.york.ac.uk/prospero/, identifier: CRD42022300908.

## 1. Introduction

In the world, hypertension affects 31.1% of adults, making it one of the most prevalent chronic illnesses (1). Type 2 diabetes, heart failure, and stroke could be secondary to hypertension (2–5), resulting in the death of patients with heart diseases and stroke, which are seriously endangering human health (6). Therefore, a novel treatment is urgently needed to control the further deterioration of hypertension. According to a meta-analysis, physical exercise helps people with hypertension lower their blood pressure (BP) (7). IMT has been shown to be a crucial component in the rehabilitation of hypertension as an innovative physical activity strategy (8).

Inspiratory muscle training (IMT) is performed using a small and special breathing device that can provide resistance for inhalation [such as PowerBreath® (9)], which, with better tolerance and efficiency, can be used as an important adjunct therapy for patients with hypertension (10). The underlying mechanism for the ability of IMT to lower BP may be the central respiratory network, and its interaction with the sympathetic nerve drive and vagus nerve activity may be affected by an increase in inspiratory muscle strength, which also promotes cardiopulmonary coupling (11). Recent research suggests (12) that IMT with a 30% MIP may be able to lower BP in people with essential hypertension, as well as isolated systolic hypertension patients whose resting BP is elevated (13). However, the impact of IMT on hypertension is still debatable. Beltrami (14) has regarded the effects of IMT on hypertension as exaggerated. It (15) shows that IMT has no obvious effects on the BP of patients with hypertension, and slow breathing is more effective than IMT in lowering BP (16). Da Silva (17) has shown that loaded respiratory training can reduce BP in hypertension, however, only two RCTs were analyzed in this literature resulting in low confidence in the results. To sum up, the effects of IMT on hypertension should be further explored.

Furthermore, studies (18) have also demonstrated that patients with hypertension can have their BP under control by monitoring hemodynamic indicators, like systolic blood pressure (SBP), diastolic blood pressure (DBP), heart rate (HR), and pulse pressure (PP). The goal of this study was to conduct a meta-analysis evaluating the benefits of IMT for individuals with hypertension in order to determine how IMT affects people with hemodynamic indices of hypertension and to provide guidance and evidence for the clinical application of IMT.

## 2. Methods

The Cochrane Cooperation Organization's (19) and PRISMA guidelines for systematic evaluation and meta-analysis (20) were followed in conducting this meta-analysis. The protocol has a PROSPERO registration (registration code CRD42022300908). This study was based on the existing published literature, therefore, ethical approval is not required.

### 2.1. Eligibility criteria

Using PICOS elements as a basis, the eligibility criteria were defined (21): (1) population: patients enrolled in clinical trials who have SBP greater than 130 mmHg and/or DBP greater than 80 mmHg (22); (2) intervention: IMT was utilized in the intervention group; (3) comparison: in the control group, a blank control, sham IMT therapy, or traditional training, were implemented; (4) outcome: mean BP or PP in mmHg, HR in bpm, and standard deviation or standard error; (5) study type: randomized controlled trials. Additionally, we excluded trials according to the exclusion criteria: (1) study types were conference abstracts, non-randomized controlled trials, retrospective studies, animal experiments, case reports, etc.; (2) participants in clinical trials who had other cardiovascular and cerebrovascular diseases or metabolic diseases; (3) inability to obtain the full text and complete data.

### 2.2. Search strategy

To locate any pertinent randomized controlled trials (RCTs), the following databases were thoroughly searched: Web of Science, the Cochrane Library, PubMed, Embase, CNKI, and Wanfang databases. The literature search was carried out without language restrictions or a beginning date until July 2022 using the following terms: “high blood pressure”, “hypertension”, “blood pressure”, “diastolic blood pressure”, “systolic blood pressure”, “systolic blood pressure”, “pulse pressure”, “heart rate”, “breathing exercise”, “inspiratory muscle training”, “inspiratory loaded breathing training”, “high-resistance inspiratory muscle strength training”, “slow load breathing training”, and “IMT.” We contacted the authors via email for additional information in cases of insufficient data, and the available data were used to run analyses if they failed to respond within 14 days.

### 2.3. Study selection

Relevant literature was searched using the following process: The first step was a preliminary screening using the title and abstract, which was then loaded into Endnote X9 software, where duplicate literature was then removed. The remaining full text of literature was carefully examined in light of the established inclusion and exclusion criteria, and any content that did not meet the standards was eliminated. The whole screening process was independently performed by two reviewers (GL and LC). The third reviewer (YZ) was ready for consultation if there were any discrepancies between the two independent reviewers.

### 2.4. Data extraction

Two researchers (SZ and SYL) independently retrieved the following information: the first author, publication year, sample size, average age, type of hypertension, gender, medication, load intensity, intervention time, training time, training frequency, respiratory rate, training equipment, data (SBP, DBP, HR, and PP) of the intervention group and control group data, time of measurement, equipment of measurement, and position of measurement. When there was insufficient data, we emailed the authors for additional information, and if they didn't reply within a week, we ran analyses using the data that were available instead.

### 2.5. Quality assessment

The quality of each study was assessed by two researchers (QZh and SZ) using a modified Jadad quality scale (23) that included four questions related to scientific rigor, such as the generation of a random sequence, randomization concealment, blind method, withdrawal number, and reasons. The highest score on the modified Jadad quality scale is 7, with 1, 2, and 3 denoting low quality and 4, 5, 6, and 7 denoting high quality (24).

### 2.6. Data analysis

The Cochrane Handbook for Systematic Reviews of Interventions was used to analyze the data. For continuous variables, the mean difference (MD) and 95% confidence interval (CI) were used to compare net changes (i.e., IMT vs. control group). For each study group and important outcome, mean and standard deviations (SD) were gathered in order to calculate the effect size. A negative effect size indicated that IMT effectively reduced SBP, DBP, HR, and PP. Using the heterogeneity chi-square test and *I*^2^ statistics, this study quantitatively discussed the heterogeneity of various studies. These relevant data results were analyzed using the Revman5.4 software. The pooled MD and the 95% CI were represented graphically using a forest plot. According to its weight in the meta-analysis, each study was represented in the plot by a square. The threshold for statistical significance was a two-sided *P* value of 0.05. As the number of included researches was lower than ten pieces of research, we did not use statistical or graphical methods to evaluate publication bias.

## 3. Results

### 3.1. Selection

**Figure 1:**
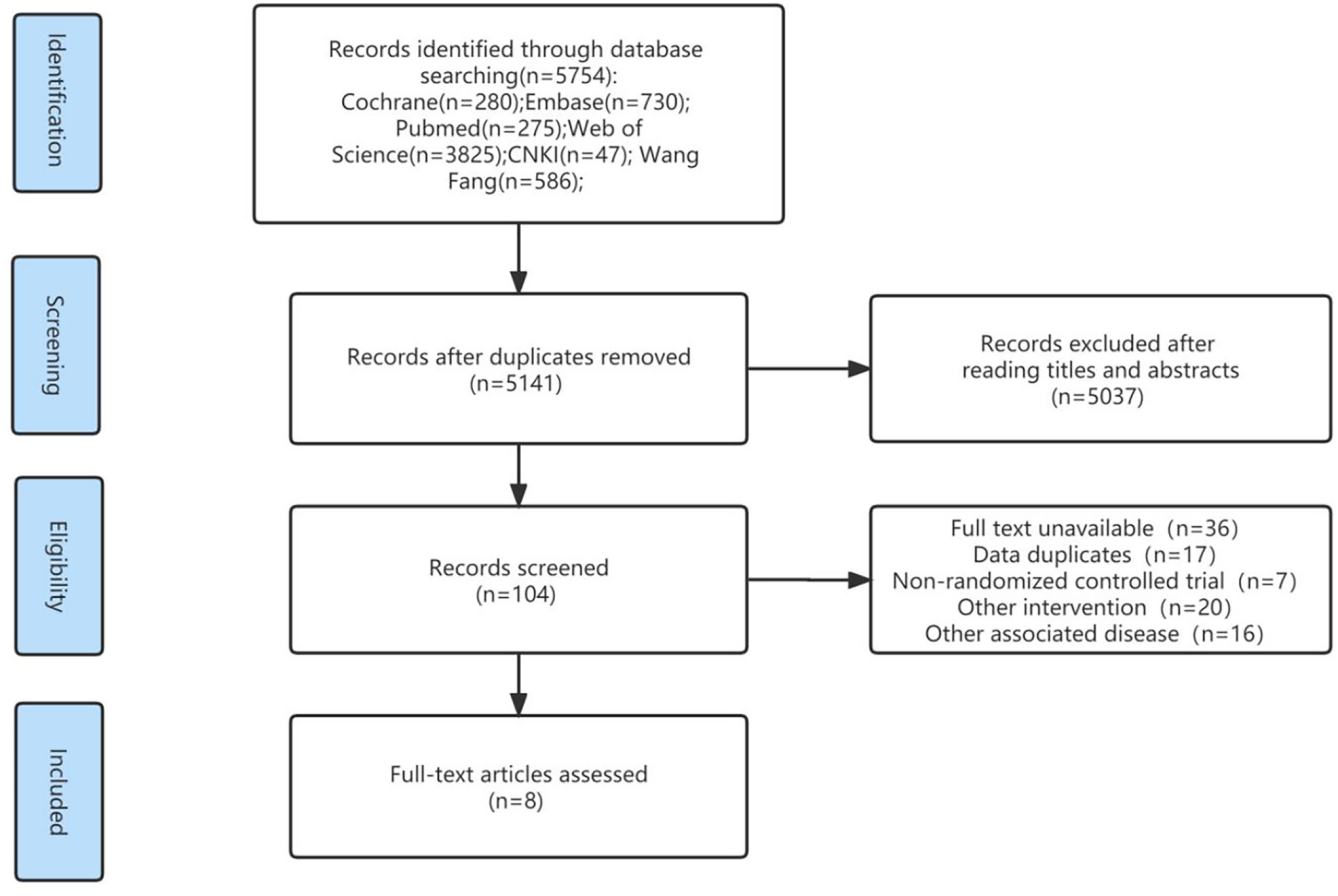
Flow chart of search retrieval and screening.

The initial search identified 5,754 pieces of literature, 5,141 of which were left after eliminating duplicate literature using the EndNote x9 software. Only 104 publications, chosen based on titles and abstracts, were used for the rest of the analysis. Eight RCTs (12, 13, 15, 25–29), seven in English and one in Chinese, were eventually included in this study after carefully reading the whole text (Figure 1). Following a full-text analysis, the main reasons for exclusion were: full text not being accessible; duplicate data; non-RCTs; other interventions; and other connected disorders.

### 3.2. Participant characteristics

Eight studies with a total sample size of 215 patients aged between 50 and 70 years were finally analyzed: seven clinical trials were designed as parallel trials (12, 13, 15, 25–29) and only one study (28) was a cross-over trial. Based on the type of hypertension, three studies (12, 15, 25) essential hypertension, three (13, 26, 27) isolated systolic hypertension, and two (28, 29) only described as hypertension. One study (28) did not record the drug information of patients, while the other seven studies (12, 13, 15, 25–27, 29) had records of drug information, mainly including angiotensin-converting enzyme inhibitors, calcium blockers, angiotensin II receptor blockers, and beta-blockers. Characteristics of the participants was shown in Table 1.

**Table 1:** Characteristics of participants.

Study	Design of trials	Sample size	Age (years)	Stage of hypertension	Gender	Medication
Craighead DH 2021 (29)	Parallel	IMST: 18 C: 18	IMST: 67 ± 2 C: 67 ± 2	Hypertension	IMST: F:9, M:9 C: F:8, M:10	Antihypertensive or other prescription medications (eg, statins)
Ferreira JB 2013 (12)	Parallel	IMT: 6 P-IMT: 7	IMT: 61.8 ± 11.1 P-IMT: 52.1 ± 8.8	Essential hypertension	IMT: F:3, M:3 P-IMT: F:5, M:2	Diuretics β-receptor blocker Calcium channel blocker ACE-l
Jones CU 2010 (25)	Parallel	LB: 10 C: 10	LB: 50 ± 5 C: 51 ± 5	Essential hypertension Stage I/II	LB: F:7, M:3 C: F:5, M:5	Enalapril atenolol hydrochorothiazide
Ublosakka-Jones C 2017 (26)	Parallel	LB: 10 C: 9	LB: 65 C: 65	Isolated systolic hypertension	LB: F:6, M:4 C: F;6, M:3	Diuretic Ang ll receptor blockers Calcium channel blockers β-receptor blocker Metformin
Ublosakka-Jones C 2018 (27)	Parallel	LB: 16 C: 16	LB: 66.4 ± 4.2 C: 68.2 ± 4.8	Isolated systolic hypertension	LB: F:8, M:8 C: F:8, M:8	Diuretics Ang Il receptors blocker Calcium channel blocker
Ublosakka-Jones C 2019 (13)	Parallel	LB: 10 C: 10	LB: 67 ± 6 C: 69 ± 3	Isolated systolic hypertension	LB: F:5, M:5 C: F:6, M:4	ACE-l Calcium channel blocker Alpha-blocker
Yuenyongchaiwat K 2019 (28)	Crossover	20	58.25 ± 13.78	Hypertension	M:50 F:50	—
Zhu K 2021 (15)	Parallel	LB: 26 C: 28	LB: 56.2 ± 8.1 C: 53.1 ± 7.9	Essential hypertension	LB: F:18, M:10 C: F:15, M: 11	Diuretic Calcium channel blocker β-receptor blocker ACE-l Ang ll receptor blockers α-receptor blocker

BMI, body mass index; LB, loaded breathing; ULB, unloaded breathing; Con, control groups; IMT, inspiratory muscle training; P-IMT, placebo-inspiratory muscle training; IMST, inspiratory muscle strength training; M, man; F, female; ACE-l, angiotensin-converting enzyme inhibitory.

### 3.3. Measurements on hemodynamic parameters

Ambulatory BP and PP were measured in two studies (12, 29) and resting BP and PP were measured before and 10 min after intervention in the other two studies (15, 28). BP and PP were measured every morning in three studies (13, 25, 27). Resting BP and PP were measured before and 8 weeks after training (26). The position of BP measurements was simply described in the patient's non-dominant arm in five articles (12, 13, 24, 26, 28). In one study, the position was depicted on the left semi-flexed arm at the heart-level height (28), but another article said the position was the patient's right upper brachial artery pressure (15). The remaining RCT indicated that this position was on each subject's arm 1 inch above the elbow over the brachial artery (26). As for measuring equipment, these studies used Oscar 2 (SunTech Medical) (29), DynaMAPA® monitor (Cardios, São Paulo, Brazil) (12), Ri-champion (Rudolf Riester GMBH Co, Germany) (13, 25, 27), BSM-6701 bedside monitor (Nihon Kohden, Tokyo, Japan) (26), Philips Intellivue MP20 bedside monitor (Nihon Kohden, Tokyo, Japan) (28), and Yuwell desktop sphygmomanometer (15) respectively. The measurement time, position, and equipment of PP and HR in 8 RCTS were also shown in Table 2.

**Table 2:** Hemodynamic parameters of participants.

Study	SBP (mmHg)	DBP (mmHg)	HR (bpm)	PP (mmHg)	Measurements		
					Time	Position	Equipment
Craighead DH 2021 (29)	IMST:131 ± 3 C:135 ± 3	IMST:75 ± 2 C:74 ± 2	—	—	ABPM was measured at baseline and after 6 weeks of IMST or sham training.	BP: the subject's non-dominant arm	BP: Oscar 2; (SunTech Medical)
Ferreira JB 2013 (12)	IMT: 133.1 ± 9.8 IMT: 130 ± 6.4	IMT: 80.6 ± 12.3 P-IMT: 86.4 ± 9.3	IMT:61.3 ± 12.7 P-IMT:71.0 ± 14.7	—	ABPM was measured before and after the interventions. HR was acquired immediately before and after the interventions.	BP: the subject's non-dominant arm HR: ECG electrodes	BP: DynaMAPA® monitor (Cardios, São Paulo, Brazil) HR: Biopac MP150 system(Biopac, California, USA)
Jones CU 2010 (25)	LB: 142 ± 8.9 ULB: 141 ± 5.9 C: 142 ± 9.6	LB:87 ± 5.2 ULB: 85 ± 4.4 C: 87 ± 5.3	LB: 75 ± 5.8 ULB:74 ± 5.6 C:73 ± 7.3	LB: 55 ± 9.4 ULB:57 ± 4.9 C:56 ± 9.8	HR, BP and PP were made in every morning between 7.00 and 9.00 am before the patients began training and in the week following the last training session.	BP/PP/HR: the subject's non-dominant arm	BP/PP/HR: Ri-champion(Rudolf Riester GMBH Co, Germany)
Ublosakka-Jones C 2017 (26)	LB: 139 ± 10 C: 139 ± 10	LB: 73 ± 13 C: 76 ± 9	LB: 68 ± 8 C: 68 ± 11	LB: 67 ± 7 C: 63 ± 8	Resting BP and HR were reported for assessment before and immediately after the 8-week training period.	BP/PP: each subject's arm 1 inch above the elbow over the brachial artery HR: ECG electrodes	BP/PP: BSM-6701 bedside monitor (Nihon Kohden, Tokyo, Japan) HR: BiopacTM SS11LA (BIOPAC System)
Ublosakka-Jones C 2018 (27)	LB: 141.5 ± 6.5 C: 141.4 ± 4.8	LB: 70.4 ± 3.2 C: 73.0 ± 7.5	LB: 71.2 ± 6.9 C: 72.5 ± 11.6	LB: 71.1 ± 6.6 C: 63.7 ± 9.7	HR, BP and PP were carried out, every morning before 9:00 am during the 2 week run-in and the following 8 week training period.	BP/PP/HR: the subject's non-dominant arm	BP/PP/HR: ri-championN (Jungingen, Germany)
Ublosakka-Jones C 2019 (13)	LB: 143 ± 9 C: 137 ± 7	LB:80 ± 8 C:76 ± 12	LB: 72 ± 13.4 C: 71 ± 13.8	LB: 63 ± 5.7 C: 76 ± 7.3	HR, BP and PP data were noted every day before 9:00 am,together with a record of their training, during the 2-week run-in,the following 8-week training period.	BP/PP/HR: the subject's non-dominant arm	BP/PP/HR: ri-championN (Jungingen, Germany)
Yuenyongchaiwat K 2019 (28)	LB: 139.15 ± 15.82 C: 138.65 ± 14.21	LB: 82.95 ± 10.48 C: 83.60 ± 9.21	LB: 80.50 ± 15.59 C: 78.35 ± 15.60	—	HR, BP and PP were performed prior to and after intervention with the 10-min resting.	BP/PP/HR: on the left semi- flexed arm at the heart-level height	BP/PP/HR: Philips Intellivue MP20 bedside monitor (Nihon Kohden, Tokyo, Japan)
Zhu K 2021 (15)	LB: 137 ± 19 C:134 ± 21	LB: 89 ± 13 C: 87 ± 11	LB: 77 ± 5 C: 74 ± 6	—	HR, BP and PP were performed prior to and after intervention with the 10-min resting.	BP: Patient's right upper brachial artery pressure HR: The pulse of the wrist	BP: Yuwell desktop sphygmomanometer HR: —

LB, loaded breathing; ULB, unloaded breathing; Con, control groups; IMT, inspiratory muscle training; P-IMT, placebo-inspiratory muscle training; IMST, inspiratory muscle strength training; SBP, systolic blood pressure; DBP, diastolic blood pressure; BP, blood pressure; HR, heart rate; ABPM, ambulatory blood pressure monitoring.

### 3.4. Intervention characteristics

Five (12, 13, 25–27) intervention groups were low-intensity IMT; two (15, 28) were medium-intensity IMT; and one study (29) was high-intensity IMT. Six studies (13, 15, 26–29) showed no intervention in the control group; one study (12) showed placebo-IMT; and another study (27) included both the control group without intervention measures and placebo-IMT. Two studies (13, 27) used the BreatheMAX device; two studies (15, 29) used the POWERbreathe device; two studies (25, 26) used a water pressure threshold bottle; one study (12) used the Threshold Inspiratory Muscle Trainer (HealthScan Products Inc., Cedgrove, New Jersey), and one study (28) used the TU-Breath Training device. The intervention duration of one (29) study was 6 weeks' one study (28) was 1 week, and the remaining six studies (12, 13, 15, 25–27) were 8 weeks. Characteristics of the intervention was described in Table 3.

**Table 3:** Characteristics of the intervention.

Study	Intensity	Duration of intervention	Training session (min)	Weekly frequency	Respiratory rate	Device
Craighead DH 2021 (29)	Week 1: 55% MIP week 2: 65% MIP week 3–6: 75% MIP	6 weeks	30 inspiratory maneuvers per day	6 days per week	30 inspiratory maneuvers (5 sets of 6, 1-min rest between sets)	POWERbreathe K3
Ferreira JB 2013 (12)	30% MIP	8 weeks	30 min	7 days per week	15 to 20 breaths/min	Threshold Inspiratory Muscle Training device
Jones CU 2010 (25)	20 cm H_2_0	8 weeks	30 min	Twice a day, 7 days a week	5 s rest after every 6 deep breaths	Water Pressure Threshold Bottle
Ublosakka-Jones C 2017 (26)	20 cm H_2_0	8 weeks	30 min	Twice a day, 7 days a week	5 s rest after every 6 deep breaths	Water Pressure Threshold Bottle
Ublosakka-Jones C 2018 (27)	25% MIP	8 weeks	60 breaths a day	7 days per week	6 breaths per minute with an inspiratory time of 4 s and expiratory time of 6 s	BreatheMAX
Ublosakka-Jones C 2019 (13)	25% MIP	8 weeks	60 breaths a day	7 days per week	6 breaths per minute with an inspiratory time of 4s and expiratory time of 6s	BreatheMAX
Yuenyongchaiwat K 2019 (28)	40% MIP	1 weeks	10 min	7 days per week	Three sets of 10 breathing exercises	TU-Breath Training
Zhu K 2021 (15)	40% MIP	8 weeks	Continuous training for 30 times was 1 group, 2 groups per day	4 days per week	–	POWERbreathe

MIP, maximal inspiratory pressure.

### 3.5. Quality assessment results including research

According to the improved Jadad scale, low quality was defined as 1–3 points and high quality as 4–7 points. Seven studies (12, 13, 15, 26–29) scored >3 points and were classified as high-quality literature, while one study (25) received 3 points and was labeled as low-quality literature. The quality of the included RCT studies was shown in Table 4.

**Table 4:** The modified jadad scores of the included studies.

First author (Year)	Randomization	Allocation concealment	Blinding	Withdrawals and dropouts	Total
Craighead DH 2021 (29)	2	2	2	1	7
Ferreira JB 2013 (12)	2	1	2	1	6
Jones CU 2010 (25)	1	1	0	1	3
Ublosakka-Jones C 2017 (26)	1	2	1	1	5
Ublosakka-Jones C 2018 (27)	1	2	0	1	4
Ublosakka-Jones C 2019 (13)	1	1	1	1	4
Yuenyongchaiwat K 2019 (28)	1	1	1	1	4
Zhu K 2021 (15)	2	1	1	1	5

### 3.6. Main results

#### 3.6.1. Effects of IMT on systolic blood pressure

**Figure 2:**
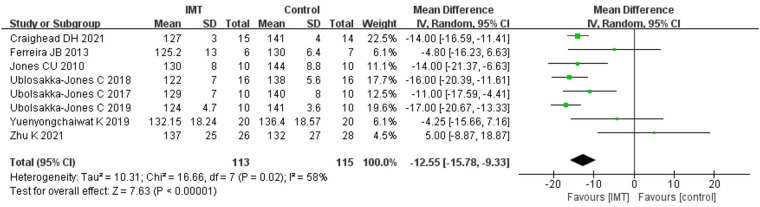
Meta-analysis forest of diagram of systolic blood pressure.

SBP after treatment was compared between the intervention group using IMT and the control group. The results demonstrated that IMT might lower the SBP of patients with hypertension; According to data analysis from seven trials using a random effect model, IMT could improve the SBP of patients with hypertension. [*n* = 228, MD = −12.55 mmHg, 95%CI (−15.78, −9.33), *P *< 0.01] (Figure 2).

#### 3.6.2. Effects of IMT on diastolic blood pressure

**Figure 3:**
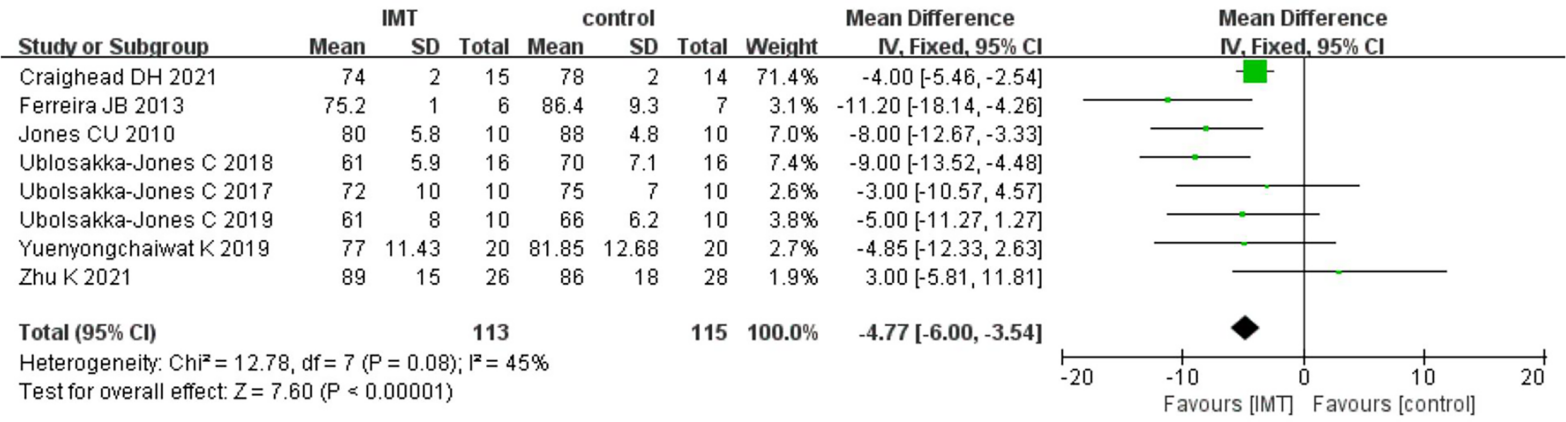
Meta-analysis forest diagram of diastolic blood pressure.

After analyzing the DBP data in the IMT and control groups, high heterogeneity was found (*I^2^* = 45%). Therefore, for the meta-analysis, we used a fixed effects model. The results demonstrated that IMT training was more successful than a control group in decreasing DBP in patients with hypertension [*n* = 228, MD = −4.77 mmHg, 95% CI (−6.00, −3.54), *P *< 0.01] (Figure 3).

### 3.7. Secondary results

#### 3.7.1. Effects of IMT on heart rate

**Figure 4:**
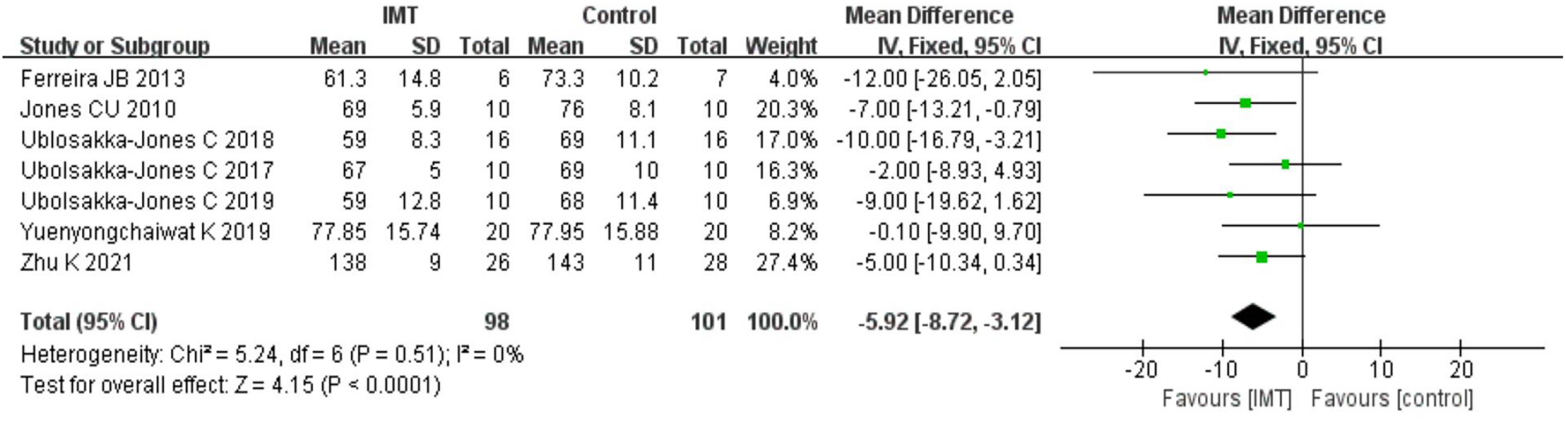
Meta-analysis forest diagram of heart rate.

Because Daniel H 2021 (29) study could not obtain relevant data, we analyzed the data from two groups of patients post-treatment in the other seven trials. According to the results of the fixed effect model, IMT had a stronger impact on lowering the HR of patients with hypertension than the control group [*n* = 199, MD = −5.92 bpm, 95% CI (−8.72, −3.12), *P *< 0.01] (Figure 4).

#### 3.7.2. Effects of IMT on pulse pressure

**Figure 5:**
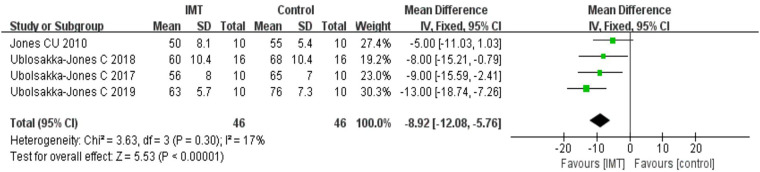
Meta-analysis forest diagram of pulse pressure.

Four studies provided results for PP with a total of 92 patients. The heterogeneity (*I^2^* = 17%) was small; thus, it was decided to use the fixed effect model, and the analysis's findings revealed that IMT could reduce the PP of patients with hypertension [*n* = 92, MD = −8.92 mmHg, 95% CI (−12.08, −5.76), *P *< 0.01] (Figure 5). As interventions in these four studies were all low-intensity IMT, the subgroup analysis was not performed.

### 3.8. Subgroup analysis

#### 3.8.1. Effects of IMT on systolic blood pressure

**Figure 6:**
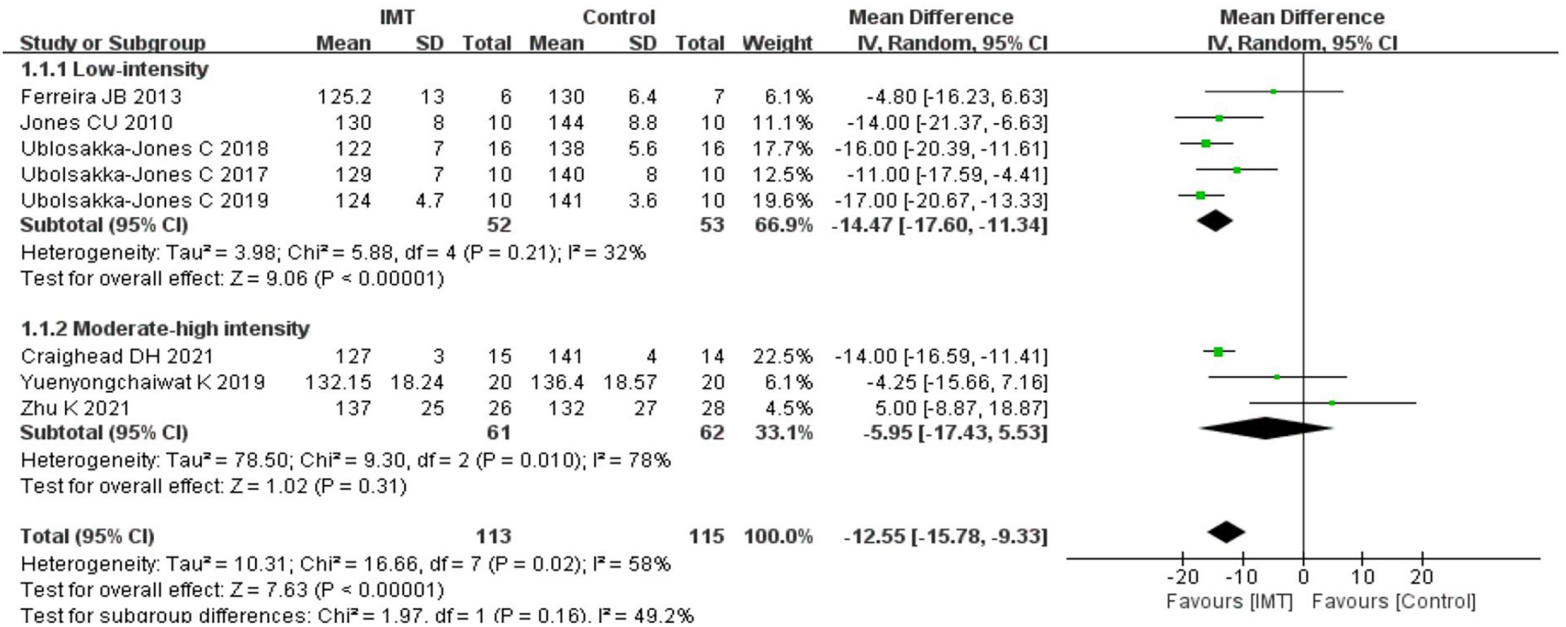
Meta-analysis forest diagram of subgroup analysis of systolic blood pressure.

About 30% of MIP is considered low-intensity IMT (30), whereas >30% MIP is medium-high-intensity IMT. Eight studies were divided into two groups: low-intensity IMT and medium-high-intensity IMT, and two subgroup analyses were conducted. Subgroup analyses showed that low-intensity IMT was effective in decreasing SBP of patients with hypertension [*n* = 105, MD = −14.47 mmHg, 95% CI (−17.60, −11.34), *P *< 0.01] (Figure 6); however, medium-high-intensity IMT had no positive effects on reducing SBP of patients with hypertension [*n* = 123, MD = −5.95 mmHg, 95% CI (−17.43, 5.53), *P* = 0.31] (Figure 6).

#### 3.8.2. Effects of IMT on diastolic blood pressure

**Figure 7:**
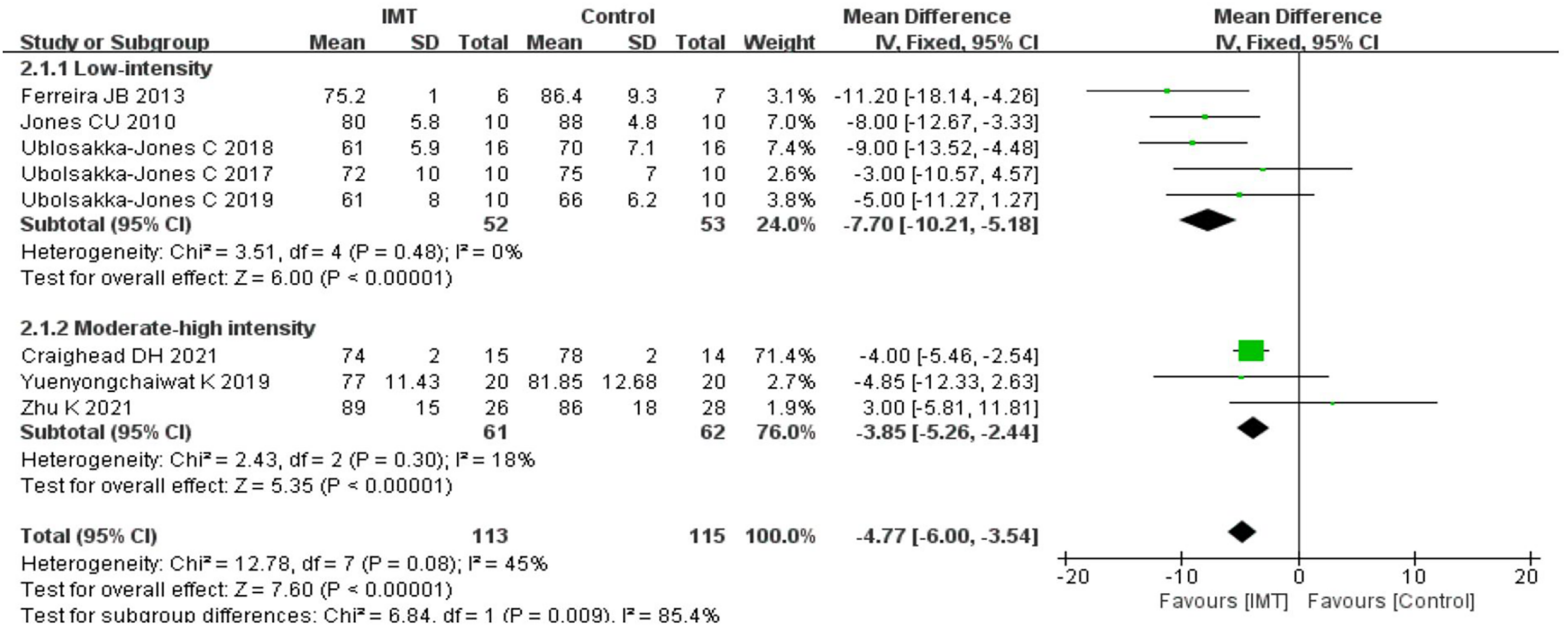
Meta-analysis forest diagram of subgroup analysis of diastolic blood pressure.

Similarly, a subgroup analysis was carried out on the DBP data from eight trials, which were separated into two groups: low-intensity IMT and medium-high-intensity IMT. Sub-group analysis showed that low-intensity IMT could improve the DBP of patients with hypertension [*n* = 106, MD = −7.70 mmHg, 95% CI (−10.21, −5.18), *P* < 0.01] (Figure 7); however, medium-high-intensity IMT did not help reduce the DBP of patients with hypertension [*n* = 123, MD = −3.85 mmHg, 95% CI (−5.26, −2.44), *P* = 0.01] (Figure 7).

#### 3.8.3. Effects of IMT on heart rate

**Figure 8:**
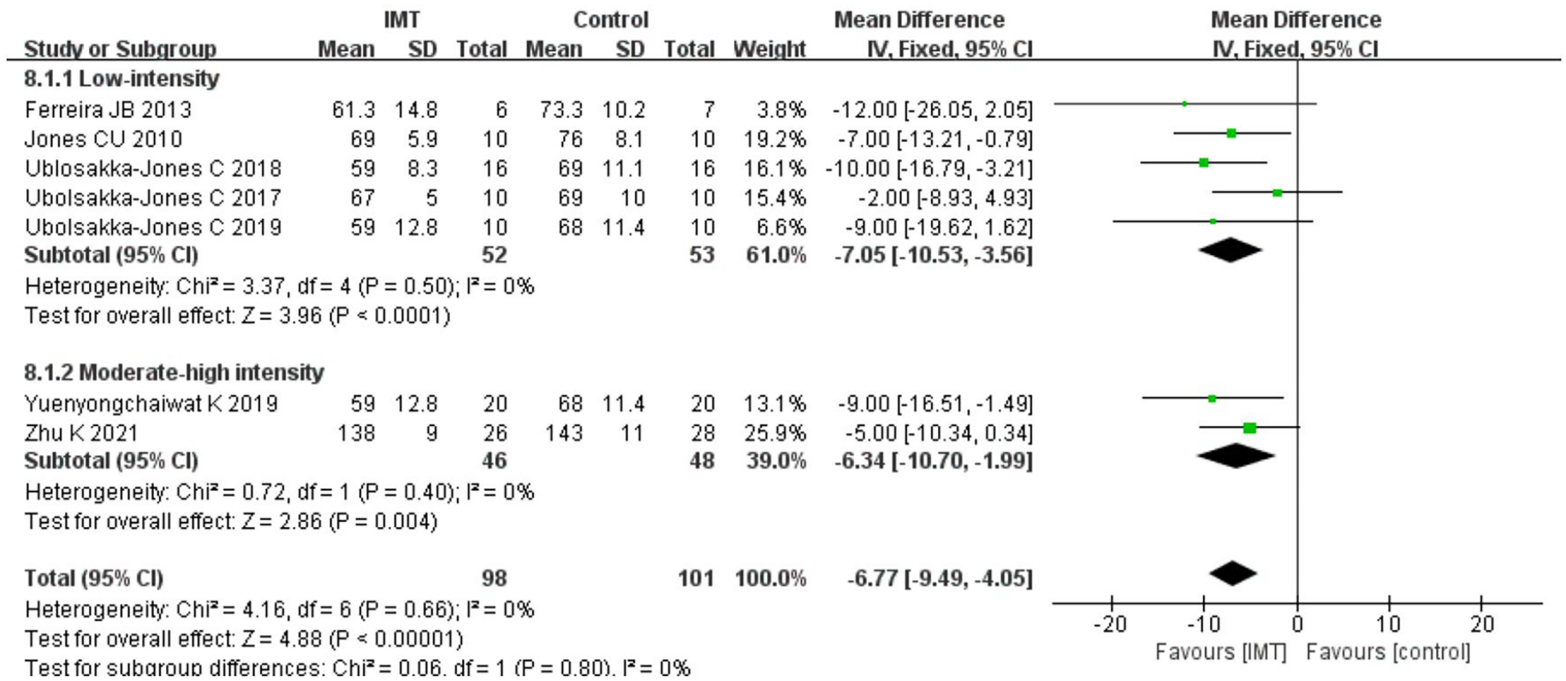
Meta-analysis forest diagram of subgroup analysis of heart rate.

Since the relevant data could not be obtained in Daniel H 2021 (29), a subgroup analysis was carried out on the remaining seven studies. According to the results of the subgroup analysis, low-intensity IMT [*n* = 199, MD = −7.05 bpm, 95% CI (−10.53, −3.56), *P* < 0.01] (Figure 8) and medium-high-intensity IMT [*n* = 74, MD = −6.34 bpm, 95% CI (−10.70, −1.99), *P* = 0.004] (Figure 8) could effectively slow down the HR of patients with hypertension.

## 4. Discussion

This meta-analysis set out to assess the impact of IMT hemodynamic indexes on hypertension. Through analysis of eight RCT items (12, 13, 15, 25–29), IMT was proven to improve the hemodynamic indexes (SBP, DBP, HR, and PP) of patients with hypertension. However, low-intensity IMT was superior to medium-high-intensity IMT in regulating BP.

Having a high prevalence and incidence of hypertension is a significant public health concern because deaths and diseases of hypertension and cardiovascular systems are closely linked (31). Physical activity is one of the treatments for patients with hypertension (7). In these meta-analyses, it could be concluded that aerobic exercise (32), isometric exercise (32), breathing-control (33), and unload respiratory muscle training (17) had the effect in reducing BP in patients with hypertension. Our meta-analysis showed that IMT, an innovative physical activity, also had an active reduction in BP which was consistent with the conclusion of the analysis showing that the antihypertensive drug was effective in SBP and DBP within 6 months (−4.2/−2.0 mmHg) (34). Among the RCTs included in this literature, seven studies (12, 13, 15, 25–27, 29) clearly indicated that patients were still receiving medication and one study (28) did not clearly indicate whether patients were receiving medication or not. Combined with the effect size of the antihypertensive drug, we speculated that IMT may further reduce BP in hypertensive patients on the basis of medication. The reason for the inconsistency between these two studies may be that fewer articles have been published on hypertension in the latter study and the heterogeneity among RCTs is high. Lowering BP is quite important in the clinic. Studies suggested that every reduction of 5 mmHg BP can significantly reduce the risk of type 2 diabetes (5), heart failure (35), and stroke (36), thus it is possible that lowering BP is a means of preventing type 2 diabetes, heart failure, and stroke. And the safety of IMT in these diseases has been established (37–39). Therefore, IMT can be used as adjunct training for hypertension to lower BP and minimize the chance of developing secondary conditions in clinic.

It is possible that IMT lowers BP through a variety of mechanisms. IMT may have decreased BP based on changes in breathing patterns and reflex mechanisms (25). For example, the respiratory muscle metaboreflex activation in patients with hypertension will be activated in advance during exercise, whereas increasing the inspiratory muscle strength of patients with hypertension through IMT can slow down the early appearance of this reflex, thus reducing the hyperactivity of peripheral sympathetic nerves, which could control the rise in BP (40). In addition, arterial stiffness is one of the important inducements to develop and maintain hypertension (41). IMT could reduce SBP in patients with hypertension, which may be related to IMT altering endothelial and smooth muscle function, thereby affecting aortic stiffness (16). Regarding DBP, respiratory resistance can raise intrathoracic pressure, lower cardiac output, and decrease systemic venous return due to pulmonary artery compression (42). As a result of the significant decrease in cardiac output, which also causes a reduction in ventricular filling, the result is a reduction in DBP. Low-intensity IMT reduced BP more than high-intensity IMT; this may be related to the greater contraction intensity of respiratory muscles in medium-high-intensity IMT, which more likely stimulates mechanoreceptors in the muscles, and muscle contraction leads to increased vasculature pressure, which results in increased BP (43).

The role of HR in hypertension has been established. Patients with hypertension frequently have an increase in HR, which has been linked to an independent predictor of poor cardiovascular and death outcomes (44). Therefore, there is a need to evaluate how IMT affects HR in individuals with hypertension. Our meta-analysis showed a result that IMT was linked to an effect size of −5.29 bpm on the HR, which was similar to the results of pursed-lip breathing (−3.85 bpm) (45) and high-intensity interval training (−2.17 bpm) (46) decreasing in HR. This reduction in HR has important clinical implications for patients with hypertension. A 10 bpm rise in HR resulted in an 8% increase in the incidence of hypertension (47), while a drop in HR was associated with a reduced cardiovascular death rate in the long-term follow-up procedure (48). IMT can regulate HR probably because it can decrease cardiac sympathetic nerve activity and increase the expression of parasympathetic nerves at rest (49). In the included studies, only four RCTs evaluated PP in hypertension before and after IMT. PP is another predictor of hypertension events that, in some cases, has a superior predictive capability to SBP or DBP alone (50, 51). Our meta-analysis discovered an improvement in PP in patients with hypertension, which is associated with the outcome of the present study that SBP decreases more obviously than DBP. Therefore, IMT may reduce HR and PP while dropping BP, minimizing the risk of unfavorable cardiovascular and fatal consequences.

It is the first meta-analysis to focus on the impact of IMT on the four hemodynamic indexes of patients with hypertension, and our analysis provides evidence for IMT as a clinical auxiliary means for improving hemodynamic indexes and decreasing the risk of some secondary disorders in individuals with hypertension. However, future research will be required to establish the most appropriate type, timing, number of sessions each week, and sessions per breath for optimal treatment outcomes. Moreover, in order to better understand the circadian behavior of BP and HR, it would also be interesting to evaluate 24-hour ambulatory BP and HR measurements.

The study limitations are mainly manifested in the following aspects: (1) a small number of RCTs were included in this investigation, and the bulk of them had modest sample sizes; therefore, the total sample size was small; (2) some heterogeneity problems encountered in this study may be due to different equipment, training frequency, training duration, intervention time, and intervention intensity; (3) since the respective number of different types of hypertension was very small in the included studies, this study did not do a subgroup analysis of distinct kinds of hypertension.

## 5. Conclusion

IMT may become an auxiliary means to improve the four hemodynamic indexes (SBP, DBP, HR and PP) in patients with hypertension. Furthermore, low-intensity IMT was preferred over medium-high-intensity IMT in subgroup analyses for regulating SBP and DBP. Both low and medium-high-intensity IMT could reduce the HR of patients with hypertension. However, the training time, respiratory rate, and training frequency of IMT, as well as the therapeutic effects on different types of hypertension, should be further explored.

## Data Availability

The original contributions presented in the study are included in the article, further inquiries can be directed to the corresponding author/s.
